# Managed care updates of subscriber jail release to prompt community suicide prevention: clinical trial protocol

**DOI:** 10.1186/s12913-023-10249-5

**Published:** 2023-11-16

**Authors:** Sarah A. Arias, Kimberly Sperber, Richard Jones, Faye S. Taxman, Ted R. Miller, Sarah Zylberfuden, Lauren M. Weinstock, Gregory K. Brown, Brian Ahmedani, Jennifer E. Johnson

**Affiliations:** 1 Butler Hospital, Providence, RI USA; 2 Department of Psychiatry and Human Behavior, Brown University, Butler Hospital, 345 Blackstone Blvd., Providence, RI 02906 USA; 3 CareSource, Dayton, OH USA; 4 Center for Advancing Correctional Excellence!, George Mason University, Arlington, VA USA; 5 Pacific Institute for Research & Evaluation, Beltsville, MI USA; 6 Curtin University School of Public Health, Perth, Australia; 7 Department of Psychiatry, University of Pennsylvania, Philadelphia, PA USA; 8 Department of Psychiatry, Henry Ford Health System, Detroit, MI USA; 9 Charles Stewart Mott Department of Public Health, Michigan State University, Flint, MI USA

**Keywords:** Suicide prevention, Managed care, Medicaid, Jail, Criminal legal involvement

## Abstract

**Background:**

Recent jail detention is a marker for trait and state suicide risk in community-based populations. However, healthcare providers are typically unaware that their client was in jail and few post-release suicide prevention efforts exist. This protocol paper describes an effectiveness-implementation trial evaluating community suicide prevention practices triggered by advances in informatics that alert CareSource, a large managed care organization (MCO), when a subscriber is released from jail.

**Methods:**

This randomized controlled trial investigates two evidence-based suicide prevention practices triggered by CareSource’s jail detention/release notifications, in a partial factorial design. The first phase randomizes ~ 43,000 CareSource subscribers who pass through any Ohio jail to receive Caring Contact letters sent by CareSource or to Usual Care after jail release. The second phase (running simultaneously) involves a subset of ~ 6,000 of the 43,000 subscribers passing through jail who have been seen in one of 12 contracted behavioral health agencies in the 6 months prior to incarceration in a stepped-wedge design. Agencies will receive: (a) notifications of the client’s jail detention/release, (b) instructions for re-engaging these clients, and (c) training in suicide risk assessment and the Safety Planning Intervention for use at re-engagement. We will track suicide-related and service linkage outcomes 6 months following jail release using claims data.

**Conclusions:**

This design allows us to rigorously test two intervention main effects and their interaction. It also provides valuable information on the effects of system-level change and the scalability of interventions using big data from a MCO to flag jail release and suicide risk.

**Trial registration:**

The trial is registered at clinicaltrials.gov (NCT05579600). Registered 27 June, 2023.

**Table:** 

**Data Category**	**Information**
Primary registry and trial identifying number	NCT05579600
Date of registration in primary registry	10–14-2022
Secondary identifying numbers	1P50MH127512-01A1 8577
Source(s) of monetary or material support	National Institute of Mental Health (NIMH)
Primary sponsor	National Institute of Mental Health (NIMH)
Secondary sponsor(s)	n/a
Contact for public queries	Sarah Arias; Department of Psychiatry and Human Behavior, Butler Hospital, 345 Blackstone Blvd., Providence, RI 02906. Phone: 401–455-6261. Fax: 401–455-6235. E-mail: sarah_arias@brown.edu
Contact for scientific queries	Sarah Arias; E-mail: sarah_arias@brown.edu
Public title	Suicide Prevention for Justice Involved Managed Care Subscribers
Scientific title	Managed Care Updates of Subscriber Jail Release to Prompt Community Suicide Prevention: Clinical Trial Protocol
Countries of recruitment	United States
Health condition(s) or problem(s) studied	Suicide risk
Intervention(s)	Intervention #1: Caring Contacts Intervention #2: Reports, Re-engagement, and Training (RRT)
Key inclusion and exclusion criteria	Ages eligible for study: ≥ 18 years old Sexes eligible for study: both Accepts healthy volunteers: no Inclusion criteria: adults (≥ 18 years), (For CC condition): CareSource subscriber, recently spent time in jail; (For RRT condition): Behavioral health (BH) providers/staff employed at a CareSource BH agency Exclusion criteria: None
Study type	Interventional Allocation: Randomized Intervention model: Factorial Masking: None Primary purpose: Health Services Research Phase III
Date of first enrollment	May 2023
Target sample size	44,000 (Caring Contacts); 120 (RRT)
Recruitment status	Recruiting
Primary outcome(s)	Suicide attempts
Key secondary outcome(s)	Inpatient and emergency department mental health care visits; Outpatient behavioral health service linkage; Number of arrests
Date and Version number	10/17/23; Version 9

## Background

Passing through jail is a marker for trait and state suicide risk. Before COVID-19, there were more than 10 million jail bookings annually in the U.S [1]. Half of those booked (40–50%) report lifetime suicide ideation or risk behaviors and 13–20% have attempted suicide [2]. Individuals are most likely to be arrested when acutely ill (i.e., manic or psychotic), and disproportionately come from groups at high risk for suicide, such as males, persons with mental health and substance use disorders, socially disenfranchised, and those who have previously engaged in suicide behaviors [3]. Compared to demographically matched people, the suicide rate is 29–58 times as high in the months after jail release from incarceration [4, 5] and 3.4–18.2 times as high in the two years after release [4–9]. Given ~ 10% of all suicides with known causes in the U.S. occur in the context of a recent criminal or legal stressor (often arrest and jail detention) [10, 11], targeting suicide risk after jail detention could have a noticeable impact on national suicide rates.

High jail admissions and discharge volumes, short jail stays, and understaffing mean that many county jails do not have capacity to coordinate care between jails and outside health agencies for suicide prevention or other health needs [12–21]. Unlike prison, jail stays are usually brief (a few days for those in pretrial status [22]) and releases are often unscheduled. Outside jail, many people with criminal-legal (CL) involvement are supported by professionals within publicly funded systems, who themselves face resource restrictions, are often unaware that their client was in jail, and may discharge the client for missing appointments. With over ~ 3,100 jails and thousands of behavioral health (BH) agencies providing services, continuity of systems to reduce morbidity and mortality is a challenge [23–28].

This protocol paper describes a randomized controlled trial (RCT) developed in partnership with CareSource, a large nonprofit managed care organization (MCO) headquartered in Ohio. MCOs like CareSource, are ideal partners to assist with jail-community service linkage because their catchment areas span multiple health and jail systems. In 2018, CareSource partnered with a justice alert company (which scans publicly available booking and release data and provides alerts when individuals are booked into or released from jail) to track jail booking/release data from jails nationally for adult Ohio CareSource members. The linking system sits within CareSource (to protect confidential health information) and automatically crosswalks CareSource’s Ohio adult Medicaid subscriber list against publicly available national booking and release data weekly. The resulting Jail-Medicaid project alerts CareSource when its subscribers pass through jail so that CareSource can reach out and link them to community healthcare services. Medicaid members were identified for inclusion in this project due to the prevalence of complex behavioral health/medical conditions that can benefit from enhanced care coordination strategies. This novel data flagging approach, made possible and generalizable by advances in biomedical informatics, addresses long-standing challenges to making care connections following jail detention.

This trial employs: (1) intersection with jail as a marker of suicide risk in the general population; and (2) CareSource’s notification system to support suicide prevention and service linkage in a way that is feasible and sustainable *at scale* within the existing healthcare and MCO infrastructure. To our knowledge, this n ~ 43,000 trial will be the largest RCT for any condition in any CL involved population to date.

## Methods

The study is part of a larger partnership with the National Center for Health and Justice Integration for Suicide Prevention (NCHATS), funded by the National Institute of Mental Health. This newly established suicide prevention center focuses on building information bridges between healthcare organizations and CL systems to identify individuals at risk for suicide and connect them to care.

### Study design

The project involves testing of two evidence-based suicide prevention practices triggered by CareSource’s detention/release notifications in a partial factorial design, a rigorous and efficient study design that does not create bias [29]. The first phase targets ~ 43,000 Ohio CareSource Medicaid subscribers released from jail over 12 months, who are either randomized to receive Caring Contact letters (CC) from CareSource or Usual Care (UC) monthly for 6 months following jail release. The second phase (running simultaneously), Reports, Re-engagement, and Training (RRT), involves ~ 6,000 of the 43,000 CareSource subscribers who were seen at one of twelve contracted BH agencies in the 6 months prior to detention. During the RRT intervention, the BH agencies will receive: (a) notifications of jail release for their existing clients as a signal for potential suicide risk, (b) instructions for re-engaging these clients in services; (c) training in suicide risk screening (Columbia Suicide Severity Rating Scale [C-SSRS [30]]) and intervention practices (Safety Planning Intervention [SPI [31]]) for providers; (d) notification to use C-SSRS for all clients and SPI when a client meets additional risk criteria per the Mental Health Research Network (MHRN) risk prediction model [32] (calculated from CareSource claims data). Participants are followed for 6 months following jail release using claims data provided by CareSource. Outcomes include:

1. Effectiveness: (a) decrease in medically treated (MT) suicide attempts (*primary*); (b) decrease in all-cause injury and poisoning; (c) increased linkage to outpatient BH services; (d) increased use of the CareSource24® Nurse Advice line (CareSource24® line); (e) decreased inpatient and ED mental health care visits; and (f) decreased return to jail detention;
2. Mechanisms: service linkage and CareSource24® line use as mediators of the effects of the intervention/s on suicide outcomes;
3. Cost-effectiveness and return on investment: (a) program cost, (b) the cost of suicide-related and overall medical care; (c) cost-effectiveness; and (d) net cost to the MCO;
4. Implementation outcomes and processes: (a) scalability; (b) sustainability; (c) feasibility, acceptability, appropriateness to providers/systems; (d) actual and suggested implementation strategies.

### Settings and data sources

CareSource is an MCO serving 1.9 million members in five states. This project focuses on approximately 43,000 of CareSource’s 800,000 Ohio Medicaid subscribers who spend at least one night in jail each year. Outcome data are extracted from the CareSource Jail-Medicaid database, which integrates continuously updated jail booking, release, demographic, and health information (e.g., care team) for CareSource’s Ohio Medicaid subscribers. Providers from 12 BH agencies in Ohio were targeted for weekly reports and training in suicide screening and prevention because they treated CareSource subscribers involved in the CL system.

All information will be treated as confidential material and will be available only to authorized research staff. All paper-based materials, if applicable, will be kept in locked files. In addition, the University research servers maintain secure environments for storing and processing clinical data that adhere to or exceed HIPAA principles. The Internal Privacy Department at CareSource will review and approve all data de-identification procedures to ensure compliance with the Safe Harbor Method as well as all internal agency protocols prior to the release of any deidentified data for research purposes. Any results reported with the project will be reported in aggregate.

### Participants and randomization

All adult (age 18 + years) CareSource Medicaid subscribers in Ohio who are released from jail over the 12-month randomization period (n ~ 43,000) are eligible for study inclusion, randomized in a 1:1 ratio to the CC intervention or UC control. A subset of the 43,000 subscribers included in the CC intervention who have been seen in one of twelve contracted BH agencies in Ohio in the 6 months prior to their jail detention are included in the RRT part of the study (~ 6,000). From each of the 12 participating BH agencies, 10 providers (for a total of 120 providers overall) will be recruited and consented to participate in the RRT Intervention. These providers potentially treat individuals that are part of the ~ 6,000 of the 43,000 subscribers passing through jail who have been seen at one of these 12 BH (i.e., mental health and/or substance use) agencies in the 6 months prior to their jail detention.

The 6-month timeframe was chosen because CareSource data indicates that these subscribers are still likely to have open cases at the BH agency (~ 85%); therefore, CareSource felt comfortable asking providers to call to try to re-engage them in care. The 12 BH agencies were randomly assigned to four cohorts of 3 agencies each, to begin the RRT intervention at Months 4, 6, 8, and 10 of the CC intervention (Table 1). We chose this stepped wedge design for the RRT intervention (vs. client- or clinic-level randomization) due to ethical concerns regarding giving providers instructions to reach out to some of their clients and not others. The stepped wedge design allows all 12 agencies to receive the RRT intervention over time, while still providing statistical controls. Individuals with records in two different agencies will be analyzed with the agency that receives RRT first, with sensitivity analyses to examine any effects on results. All study procedures were performed in accordance with the Declaration of Helsinki. Policies and procedures were reviewed and approved by the Butler Hospital Institutional Review Board. Informed consent is being obtained for the focus groups and RRT intervention components. For the CC intervention, due to the retrospective nature of the data and the large population, written or verbal consent is not feasible, so a waiver of consent was granted by the Butler IRB.

**Table 1:** Study stepped wedge design

		**Study Month**				
		**1–3**	**4**	**6**	**8**	**10–12**
**Cohort**	**1**		**START**			
	**2**			**START**		
	**3**				**START**	
	**4**					**START**

### Suicide prevention interventions

#### Caring contacts

CC is an evidence-based approach that has effectively and cost-effectively reduced suicide deaths, suicide attempts, and suicidal ideation in emergency department (ED), inpatient, and veteran populations [33–41]. CC is a light-touch, highly scalable intervention, which fits well with the goals and available resources of an MCO. CC involves sending individuals brief, non-demanding messages of care [33, 34, 37, 38]. Messages: (1) include a simple expression of care or concern; (2) are non-demanding; and (3) include a reminder of available resources/ways to connect with care (see Table 2 for examples). Although CC is often used following a care episode [37, 42, 43], it can also be used to engage individuals in care for the first time [44, 45]. CC can help engage individuals who are difficult to engage in care [33]. This study is the first RCT to use CC in a CL involved population.

**Table 2:** Caring contact message content^*^

**Message 1**: *Dear [Name], Sometimes we need someone to have our back. We are here for you if you need anything – you matter to us*.
**Message 2**: *Dear [Name], We thought that we would reach out to check in. We care about your well-being. Here are some FREE resources that might be helpful*.
**Message 3**: *Dear [Name], Sometimes we need to know that there’s someone looking out for us. Know that we care about you and are here to support you*.
**Message 4**: *Dear [Name], We would like to reach out and let you know that we are here. You might find the following numbers helpful. We are always happy to hear from you*.
**Message 5**: *Dear [Name], Just a quick note to say “hi.” We care about you and would like to let you know that we are here if you need anything. You matter to us*.
**Message 6**: *Dear [Name], Hi – We are just checking in. We care and we’re here for you if you need us. We are always here if you have questions about your well-being*.

^*^Letters mailed monthly for 6-months; letters do not mention arrest or specific mental/general health treatment. All letters contained resources for the CareSource24® Nurse Advice line and the general help line (e.g., coverage questions, ID card)

To tailor messages toward individuals who have spent time in jail, we collaborated with stakeholders to design the CC messages, including individuals who spent time in jail in the past year, BH providers, and those within the community who engage with individuals who have spent time in jail. Thirty-two people in three separate virtual focus groups participated in the design and editing of the CC letters. Feedback included valuable insights into how specific wordings and phrases affected individual emotional and motivational responses to the developed messages. For instance, avoiding terms like “reminder” (i.e., too much like a reprimand) and “best wishes” (i.e., most people were not in a good place and this seemed insincere) were consistent comments from participants. Letters also were strongly preferred over postcards by participants. This type of feedback was critical for developing the caring, yet non-demanding messages in our CC letters.

CareSource chose to send CC by mail (vs. email or text) because it was the most feasible within their existing infrastructure. Jail detainees spend a short period of time in jail (typically days), so CareSource is more likely to have viable physical addresses for them. Previous studies on VA patients have shown that they prefer to receive CC messages by mail (vs. text or email) [38], and find a monthly mailing schedule acceptable [33, 34, 37, 38].

CareSource mails the finalized, approved CC letters to subscribers randomized to CC (~ 21,500) monthly for 6 months after automatic notification of jail release. The letters contain similar messages, but the wording for each is slightly different. Letters do not mention anything about the individual having spent time in jail or having received any specific services (e.g., mental health, substance use). They simply say that CareSource is here and available to help if the participant decides to reach out. CC messages list the CareSource24® line phone number (which is answered by CareSource nurses, who are trained to assess and connect callers to the appropriate services). This team works with individuals to help them navigate the healthcare system to get the most appropriate type and level of care when they need it, facilitate referrals to other internal CareSource resources to address urgent healthcare needs and/or social determinants of health, and facilitate connection to crisis lines/service. Individuals who are re-arrested during the 6-month follow-up period will continue to receive letters, but are not re-randomized.

#### Reports, re-engagement and training

##### Reports and re-engagement

During the months BH agencies are assigned to the RRT intervention condition, they receive weekly reports from CareSource with notifications of jail booking/release dates of any client seen by the agency in the 6 months prior to arrest. These reports were added to an existing, automated CareSource system that sends jail booking/release data to their contracted BH agencies. Reports include instructions for how to reengage the client and a reminder that recent time in jail is a marker for suicide risk and that the C-SSRS can be used for suicide risk assessment. Agency staff are expected to reach out to clients in a non-demanding way (“How can we help?”) and check in about suicide risk. Providers are not required to use any particular screening or intervention tool; however, they are being trained in evidence-based suicide risk screening (C-SSRS [30]) and intervention (SPI [31]) practices. Subscribers meeting additional risk criteria based on the MHRN suicide risk prediction model [29] (calculated using CareSource claims data) are flagged for additional outreach and action, including use of the C-SSRS and suggesting SPI as an option for clients with very high risk.

##### Training

Agencies assigned to the RRT intervention receive a one-day, virtual training in: (1) elevated suicide risk among individuals leaving jail detention; (2) how to use the notifications of jail release to reach out, check in with clients, and ask them if they would like to resume behavioral health care without pressuring them; and (3) evidence-based suicide risk assessment (C-SSRS [30]) and intervention (SPI [31]). SPI is the only suicide prevention intervention that has been tested in an RCT for individuals leaving jail [46]. Training in assessment and intervention include both instruction and live practice (role plays).

### Intervention fidelity

For the CC intervention, CC letters sent, the dates they were sent, and any letters returned are tracked using CareSource’s automated mailing system. Number of calls to the CareSource24® line are tracked for both CC and UC subscribers. For the RRT intervention, we can electronically track whether reports go out and whether they are timely/accurate. We are able to assess whether subscribers re-engaged in care using claims data. In addition, although there is not a scalable way to track outreach attempts for 6,000 subscribers in the RRT intervention, we meet monthly with CareSource and intervention agencies, hear their experiences, offer feedback, and help problem-solve challenges. Our team will keep structured implementation process notes from these meetings for further analysis of outreach attempts.

### Statistical power

Power analysis used a simulation study with variability in release dates, numbers eligible, and introducing a small intra-cluster correlation (ICC = 0.01). We assumed 43,000 eligible subscribers for the CC intervention with 50% randomly assigned to CC. Based on previous data, we assumed 6,000 eligible subscribers for the RRT intervention, and that 10% of the 6,000 (n ~ 600) CareSource subscribers would receive an additional risk flag based on the MHRN suicide risk algorithm. We assumed an overall six-month risk of suicide attempt of 0.05 (0.034 for most, 0.204 for those with the additional MHRN risk flag, odds ratio [OR] = 5). We ran the simulation model 2001 times. Significance was assessed using a two-tailed type-I error level of 5%. For the CC intervention, we will have 85.6% power to detect an effect equivalent to an OR of 0.89. CC has been found to have much stronger effects in previous studies [41] suggesting that we are adequately powered for CC. For the RRT intervention, if we assume an intervention effect of 0.87 OR, we will have 88.3% power to detect this effect. For subscribers receiving an additional risk flag (who will be suggested to receive SPI), we will have 96.4% power to detect an OR of 0.63 [10]. Having superadequate power for this test offsets concerns about providers potentially not providing SPI as instructed in this large, real-world study.

### Measures

Twelve months of historical (i.e., prior to arrest) and six months of prospective (i.e., after release) jail and Medicaid claims data will be extracted from CareSource’s Jail-Medicaid database for each participating subscriber for all measures, including demographics, diagnoses, suicide attempts, service use, number of arrests, dates of jail bookings/releases, and days incarcerated.

#### Primary outcome

The primary outcome is a decrease in the total number of MT suicide attempts during the 6 months following jail release. In addition to a risk prediction model (described above), the MHRN also has a well-validated algorithm for extracting MT suicide attempt information from claims data [29, 46–58], which will be used to assess the primary outcome. This method does not capture all suicide attempts, but it was designed and validated to make system- and state-wide projects such as this feasible.

#### Secondary outcomes

Our secondary outcomes include a decrease in the number of all-cause injury and poisoning events (capturing overdoses, etc. of unclear intent) extracted from claims data using ICD-10 codes [29, 46–58]; an increase in the number of outpatient BH (i.e., mental health or substance use) visits extracted from claims data; fewer mental health inpatient hospitalizations and ED visits extracted from claims data using MHRN algorithms [29, 46–57, 59]; increased use of the CareSource24® line; and a lower number of arrests 6 months after the start of the study when compared to the 12 months prior, extracted from the continuously updating Jail-Medicaid database.

#### Additional outcomes

##### Mechanisms

We will assess service linkage (y/n and number of outpatient BH visits) and reaching out for help (y/n and number of calls to the CareSource24® line) as mechanisms of effects of interventions on suicide outcomes.

##### Cost-effectiveness

Grant accounting will capture the costs of the CC mailings. Treatment received (split into suicide-related and overall behavioral health care and other medical care) as part of intervention and UC conditions (outpatient, inpatient, emergency department, BH visits) will be tracked using claims data. Costs of UC will be computed as CareSource payments plus co-pay and deductibles. We will add amortized training costs for providers but exclude other research costs that would not be incurred if RRT was standard care. The primary cost-effectiveness (CE) measure, computed from a societal perspective for each intervention arm, will be the program cost net of any change in other medical costs divided by the sum of MT suicide attempts prevented. We will also assess the net cost of the program from a MCO perspective, excluding patient payments from costs.

##### Implementation outcomes and processes

This project focuses on sustainability and scalability using the IHI Framework for Going to Full Scale [60, 61] as an implementation mode. In addition to assessing cost and cost-effectiveness as implementation outcomes, we are assessing, maximizing, and optimizing intervention: (1) scalability (per the Intervention Scalability Assessment Tool [62]); (2) sustainability (per the Program Sustainability Assessment Tool [63, 64]) (3) feasibility, acceptability, and appropriateness to providers and systems (per the Acceptability of Intervention Measure [65], Intervention Appropriateness Measure [65], and Feasibility of Intervention Measure [65]); and (4) actual and recommended implementation strategies (via process mapping and implementation process/case notes). These activities will be used to develop future scale-up focused implementation approaches to maximize scale-up and spread.

### Data analysis

Prior to final analysis and masked to intervention condition/s, we plan to examine distributions of outcome and control variables to determine appropriate functional forms to maximize explained variation.

#### Missing data

The primary source of missing data is subscribers dropping off (and possibly re-enrolling) in CareSource Medicaid plans. We aim to examine patterns of missing data and attempt to characterize the probability of missingness as a function of observed variables. We use methods of multiple imputation to address missing values, a recommended approach in the context of randomized controlled trials [66], generating 20 imputations. Baseline and interim (e.g., 4 week) values of outcome variables and pre-specified covariates are used in the imputation models, following best practice recommendations [67].

#### Effectiveness analyses

Our descriptive analysis follows the framework that is widely accepted in the literature [9]. Overall and for each intervention condition, we present the number of people followed, the number of reincarcerations, the days of non-incarcerated exposure tracked, the number and crude rate of fatal or MT suicide acts, and a relative risk computed as that crude rate divided by the crude rate for the UC group.

The main effectiveness analysis is planned as a comprehensive model (i.e., including all 43,000 subscribers) evaluating CC and RRT intervention effects, which provides optimal power and the ability to test interactive effects of interventions. All analyses will covary baseline (i.e., past year) values of dependent variables, and will use days incarcerated in the 6 months after the index jail release as an offset. The main analysis framework will be logistic regression with the cumulative risk of claims-data-identified suicide attempts requiring medical treatment over six months post-release as the outcome. Secondary analyses will include: (1) a negative binomial model and a count outcome for the number of MT suicide attempts and (2) a survival model with time to first MT attempt. Our models will include robust standard errors due to clustering at the agency level. We will adjust for study month, and include indicator variables reflecting: (1) CC intervention condition (i.e., CC or UC); (2) inclusion in the RRT intervention; (3) RRT intervention condition (i.e., RRT or Control). Identical analyses will be conducted for the other outcomes. We will evaluate the interaction of CC and RRT interventions by including an interaction term of the two indicators.

#### Moderators

We will test sex, race/ethnicity, past suicide attempt (y/n), past 6-month BH visit (y/n), arrest in 12 months prior to the index arrest (y/n), cumulative days of incarceration during the study period, area Deprivation Index [68, 69], Mental Health Professional Shortage Area (HPSA) score [70, 71], and per capita incarceration (by zip code) as moderators of intervention effects on suicide attempts.

#### Mechanism analysis

We will examine number of outpatient BH claims in the 6 months following the index jail release and number of calls to CareSource24® line as mediators of the intervention/s effects on MT suicide attempts by adding this variable to our effectiveness model. We will use bootstrap methods to estimate 95% uncertainty intervals around the mediated effect.

#### Cost-effectiveness & return on investment

Costs (and savings) in future years will be discounted to present value in the year of jail release [72]. We will bootstrap the 95% uncertainty interval around the CE ratios. Analyses from the MCO perspective will compute the change in claims costs by running a generalized linear model (GLM) with gamma, inverse Gaussian, or Poisson variance based on data distribution, the log link function, and robust standard errors [14]. We will use a 2-part GLM unless less than 5% of patients have zero costs. Independent variables will include treatments received, demographics, Elixhauser co-morbidities [15], and days of MCO coverage after jail discharge. We also will track the MCO separation rate post-discharge from jail by group.

### Data and safety monitoring

The study investigators are responsible for implementing and maintaining quality assurance and quality control systems for this study. The protocol was reviewed and approved by the relevant IRBs prior to study start. In addition, this study utilizes a central data and safety monitoring board (DSMB), who also reviewed and approved the study protocol. Standard reporting and monitoring “adverse events” as typically defined (i.e., patient death, hospitalization, etc.) in real time is not possible because: (1) claims data lag, and (2) data are aggregated and de-identified. The DSMB will review extant safety data at 6, 9, and 12 months after the study start.

## Discussion

In line with the larger NCHATS goals, the current study aims to leverage data linkage for suicide prevention at points of contact with the CL system. When completed, this will be the largest RCT for any condition in any population that is CL involved of which we are aware**.** This study will also be the first to evaluate MCO-provided flags for BH re-engagement and suicide prevention services for recently released individuals, a large population that contributes significantly to U.S. suicide rates. Involving MCOs, who have the capacity to work on a large scale, is critical, given the sheer volume of jail detentions per year. CareSource offers the first and best case of generalizable big health-CL data linkage; few systems have this type of data linkage, and they have figured out a way to do data linkage that is generalizable to other systems and does not rely on special relationships with the jails. It will also demonstrate how to use communication to improve public health outcomes. We believe this study will provide the first assessment of the utility of that linkage and provide a replicable model for future studies. Because these algorithms and approaches are generalizable to other systems (i.e., they do not rely on platform-specific compatibilities), we can demonstrate how notification of CL contacts can be leveraged for community suicide prevention and, by extension, other health conditions, to rapidly advance the field. This study will also be the first to evaluate CC as a suicide prevention method for individuals released from jail.

Given jail detention is a marker for suicide risk, regular information to Medicaid MCOs about their subscribers’ jail bookings/releases can serve as a catalyst for identification and preventative action (i.e., suicide prevention, treatment engagement). Our approach addresses the National Association of Counties goal to better serve CL involved individuals with mental health problems in the community [73, 74] and the National Action Alliance for Suicide Prevention’s goal to reach individuals other than standard care seekers [75]. This is the first RCT in which an MCO takes a lead role in addressing and improving suicide outcomes for subscribers recently released from jail. It also illustrates the value of a jail-MCO data linkage as a means of providing interventions. Partnering with MCOs to bridge jail and community care could produce life-saving linkages. Involving end-users intimately in study and intervention design, as we have done in this proposal, helps shorten the research-to-practice pipeline [56].

## Data Availability

As part of the P50 NCHATS infrastructure, relevant de-identified data collected as part of this research project will be deposited into the NIMH Data Archive (NDA).

## Abbreviations

BH: Behavioral health
CC: Caring contacts
CE: Cost-effectiveness
CL: Criminal-legal
C-SSRS: Columbia Suicide Severity Rating Scale
DSMB: Data and safety monitoring board
GLM: Generalized linear model
IRB: Institutional review board
MCO: Managed care organization
MHRN: Mental Health Research Network
MT: Medically-treated
NCHATS: National Center for Health and Justice Integration for Suicide Prevention
RCT: Randomized controlled trial
RRT: Reports, re-engagement, and training
SPI: Safety Planning Intervention
UC: Usual care
